# Novel Predictors of COVID-19 Protective Behaviors Among US Adults: Cross-sectional Survey

**DOI:** 10.2196/23488

**Published:** 2021-04-20

**Authors:** Ken Resnicow, Elizabeth Bacon, Penny Yang, Sarah Hawley, M Lee Van Horn, Lawrence An

**Affiliations:** 1 Center for Health Communications Research Rogel Cancer Center University of Michigan Ann Arbor, MI United States; 2 Department of Health Behavior & Health Education School of Public Health University of Michigan Ann Arbor, MI United States; 3 Division of General Medicine School of Medicine University of Michigan Ann Arbor, MI United States; 4 VA Center for Clinical Management Research VA Ann Arbor Healthcare System Ann Arbor, MI United States; 5 Department of Psychology University of New Mexico Albuquerque, NM United States

**Keywords:** COVID-19, protective behavior, psychological predictors, reactance, conspiracy beliefs, public health, health communication, communication, protection, behavior, psychology

## Abstract

**Background:**

A central component of the public health strategy to control the COVID-19 pandemic involves encouraging mask wearing and social distancing to protect individuals from acquiring and transmitting the virus.

**Objective:**

This study aims to understand the psychological factors that drive adoption or rejection of these protective behaviors, which can inform public health interventions to control the pandemic.

**Methods:**

We conducted an online survey of a representative sample of 1074 US adults and assessed three novel potential predictors of COVID-19 behaviors: trait reactance, COVID-19 conspiracy beliefs, and COVID-19 apocalypse beliefs. Key outcomes (dependent variables) included an index of COVID-19 protective behaviors, the number of trips taken from the home, and COVID-19 knowledge.

**Results:**

In bivariate analyses, all three predictors were significantly correlated in the hypothesized direction with the three COVID-19 outcomes. Specifically, each predictor was negatively (*P*<.01) correlated with the COVID-19 protective behaviors index and COVID-19 knowledge score, and positively correlated with trips taken from home per week (more of which was considered higher risk). COVID-19 protective behaviors and COVID-19 knowledge were significantly lower in the top median compared to the bottom median for all three predictors. In general, these findings remained significant after adjusting for all novel predictors plus age, gender, income, education, race, political party, and religiosity. Self-identified Republicans (vs other political affiliations) reported the highest values for each of the novel predictors.

**Conclusions:**

This study can inform the development of health communication interventions to encourage the adoption of COVID-19 protective behaviors. Interestingly, we found that higher scores of all three novel predictors were associated with lower COVID-19 knowledge, suggesting that lack of an accurate understanding of the virus may be driving some of these attitudes; although, it is also possible that these attributes may interfere with one’s willingness or ability to seek and absorb accurate health information. These individuals may be particularly immune to accepting new information and yielding their beliefs. Health communication professionals may apply lessons learned from countering similar beliefs around climate change and vaccine hesitancy. Messages designed for individuals prone to reactance may be more effective if they minimize controlling language and emphasize the individual’s independence in adopting these behavioral recommendations. Messaging for those who possess conspiracy beliefs should similarly not assume that providing evidence contrary to these beliefs will alone alter behavior. Other communication techniques such as *rolling with resistance*, a strategy used in motivational interviewing, may be helpful. Messaging for those with apocalyptic beliefs may require using religious leaders as the message source and using scripture that would support the adoption of COVID-19 protection behaviors.

## Introduction

A central component of the public health strategy to control the spread of the COVID-19 pandemic and the associated morbidity and mortality is to encourage behaviors that protect individuals from acquiring and transmitting the virus. Key protective behaviors that have been recommended by US and global health organizations such as the World Health Organization and the Centers for Disease Control and Prevention include consistent wearing of a facial mask, social distancing, handwashing, and avoiding large gatherings [1]. Until a vaccine or more effective treatments become widely available, behavior change will remain the core of the public health strategy.

Understanding individual-level attributes that are associated with adoption of these behaviors is critical to controlling the spread of COVID-19. To date, most of the research on COVID-19 protective behaviors has focused on demographic variables such as gender and race as well as social cognitive variables such as perceived risk and knowledge regarding the virus [2-5]. Less attention has been given to personality factors and constructs beyond the traditional models of health behavior (eg, perceived risk). Understanding the psychological traits that drive adoption of these protective behaviors can inform social marketing campaigns and behavior change interventions.

To address these gaps in understanding about what drives individual protective behavior choices, we identified three novel predictors based on both theoretical and empirical considerations. Psychological *reactance* theory, originally proposed by Brehm and Brehm [6] posits that when an individual’s sense of behavioral freedom is threatened, the individual is motivated to restore the perceived loss of freedom by psychologically and behaviorally rejecting the behavior, even if the behavior may be in their best interest. *Conspiracy beliefs* can be defined as unsubstantiated, implausible assertions that malevolent and hidden forces control our social institutions, and these nefarious forces secretly plot major events such as 9/11; covering up alien visitations; and, in the case of this study, the creation and spread of COVID-19. Often these beliefs reject other simpler explanations that are more probable and plausible [7]. Conspiracy beliefs have previously been found to be associated with lower adoption of protective behaviors such as vaccination and condom use [7-11]. With regard to COVID-19, a few studies have found a negative association between conspiracy beliefs, both measured as a global trait and specific to COVID-19, and positive attitudes toward and adoption of COVID-19 protective behaviors [4,10,12-14]. Finally, we were interested in the potential impact of *apocalyptic beliefs*. Apocalypticism is the generally religious belief that the end of the world is imminent [15], and civilization will soon come to a tumultuous end due to some catastrophic global event such as war, famine, or disease and more recently global warming and the COVID-19 pandemic. These beliefs often include some sense of divine punishment for immorality or disobedience and spare the *righteous* who obey God’s rules. For some Christians, these beliefs include the rapture, when both living and dead believers will ascend in to heaven to meet Jesus Christ at the Second Coming. Our underlying assumption is that individuals who believe in the apocalypse will be less likely to adhere to public health recommendations around COVID-19, in part because they welcome the end of days and the Second Coming of Christ. Although we could find no studies reporting the association between apocalyptic beliefs and COVID-19 protective behaviors, conceptual and empirical work has shown that such beliefs may impact behaviors related to climate change and violence [16,17], and we suspected it might play a role in the adoption of COVID-19 protective behaviors.

We conducted a national online survey and report here the association between these three potential novel predictors—(1) trait reactance, (2) COVID-19 conspiracy beliefs, and (3) COVID-19 apocalypse beliefs—and COVID-19 protective behaviors.

## Methods

### Sampling

Surveys were completed online using a sample provided by Dynata [18] between May 19-22, 2020. Dynata’s research panel comprises an opt-in list of over 60 million individuals worldwide. For this study, we requested a nationally representative sample of 1000 US adults 18 years or older. Quotas were used to approximate national rates for age, gender, race, income, and region for the overall US population. Our survey was conducted as open enrollment, whereby eligible panel members who log in to the Dynata website were offered a chance to partake in this survey. Surveys were completed using the Qualtrics online platform. Participants received a modest payment from Dynata for completing their survey. Dynata incentives vary based on individual preferences and include cash, frequent traveler or customer loyalty points, or a donation to a charity. The reward value is based on the amount of effort required and the population surveyed. Regardless of the type of incentive, the value is the same for every respondent in a given study. In this study, the value was US $1.00. The full survey assessed a range of individual and household characteristics and attitudes and behaviors related to the COVID-19 pandemic as well as demographics. Other than screener items, no survey items were required to progress (ie, no strict validation was used). After excluding implausible values (<10 minutes and >2 hours), the mean minutes to complete the survey was 25.3 (range 10.1-117.1) minutes.

A total of 2272 individuals clicked on the invitation link, 187 did not complete the age screener or consent, and 609 were ineligible or refused consent. This yielded 1476 surveys from age-eligible consenting individuals. To ensure the quality of the respondent data, we excluded 402 of the 1476 surveys based on two criteria. First, we excluded 375 surveys from individuals who completed the full survey in less than 10 minutes. We considered 10 minutes the minimum time required to complete a valid survey. Second, we excluded 27 surveys for individuals who answered all items within a 16-item block of items assessing attitudes toward the pandemic with an identical response. This is the equivalent of clicking down an entire column (eg, all strongly agree or disagree) for all items. Because some of the 16 items in this section were worded in the positive direction (eg, social distancing has slowed the spread of COVID-19) and others in the negative direction (eg, social distancing is not really doing much good), we considered these *response set* patterns contradictory and therefore an indication that the validity of that survey was suspect. After applying these exclusions, 1074 surveys remained for the present analyses.

### Measures

#### Primary Predictor Measures (Independent Variables)

##### Trait Reactance

We selected 5 items from the widely used Hong Reactance scale [19-23]. The scale measures trait reactance rather than reactance specific to COVID-19 recommendations. Each item was answered along a five-point continuum ranging from strongly disagree to strongly agree. Internal consistency in our sample was 0.87. The five items, averaged to create a mean scale, were:

1. I become angry when my freedom of choice is restricted.
2. Regulations trigger a sense of resistance in me.
3. When something is prohibited, I usually think, “That’s exactly what I am going to do.”
4. It disappoints me to see others submitting to society’s standards and rules.
5. Advice and recommendations usually induce me to do just the opposite.

##### COVID-19 Conspiracy Beliefs

We developed a brief, three-item scale based on prior studies of COVID-19 and other health issues [7,14]. The scale is intended to measure conspiracy beliefs regarding COVID-19 rather than a generalized conspiracy trait or worldview [14]. Each item was answered along a five-point continuum ranging from definitely false to definitely true. Internal consistency in our sample was 0.74. The three items, averaged to create a mean scale, were:

1. The real truth about COVID-19 is being kept from the public.
2. People in power are using COVID-19 as an excuse to monitor and control the public.
3. The media is making COVID-19 seem more dangerous than it really is.

##### COVID-19 Apocalypse Beliefs

We developed a new brief scale informed by theological definitions and prior related work [15,17]. Each item was answered along a five-point continuum ranging from strongly disagree to strongly agree. Internal consistency in our sample was 0.92. The three items, averaged to create a mean scale, were:

1. The COVID-19 pandemic is a sign that the apocalypse is coming.
2. The COVID-19 pandemic is a sign that Jesus will soon be returning.
3. The COVID-19 pandemic is a sign that the rapture is coming.

#### Outcomes Measures (Dependent Variables)

##### Adoption of Positive COVID-19 Protection Behaviors

We examined the frequency of five self-reported behaviors over the past week, all of which are recommended for reducing the risk of transmitting or acquiring COVID-19 [1]. For each item the responses were: rarely or never (coded 1), some of the time (coded 2), most of the time (coded 3), almost all of the time (coded 4), and all of the time (coded 5). The values of 1-5 for each item were summed to form an index score with a range of 5-25. The alpha value for the five behaviors was .84 in this sample.

1. Staying home as much as possible.
2. Wearing a mask or face covering when I go out of the house.
3. Staying at least 6 feet (about 3 steps) away from people I do not live with.
4. Avoiding gatherings or groups of other people.
5. Keeping my hands clean.

##### COVID-19 Knowledge

We created a seven-item scale, with each item answered definitely false to definitely true. A response was coded as correct by answering definitely or probably false for items 4, 5, 6, and 7, and definitely or probably true for items 1, 2, and 3. Correct scores were summed, yielding a total score from 0 to 7. Internal consistency for the seven items was 0.77.

1. A vaccine is not yet available for COVID-19.
2. COVID-19 can be easily spread from one person to another.
3. Many thousands of people have died from COVID-19.
4. Most people already have immunity to COVID-19.
5. Symptoms of COVID-19 are always visible.
6. There are effective treatments for COVID-19 that can cure most people.
7. Having COVID-19 is about as dangerous as having the flu.

##### Trips Leaving the Home

We assumed that a higher number of trips from home indicated higher risk behavior. We queried leaving the home in the past week across various types of trips. For each, we asked, “In the last seven days, how many times did you go out of your home for each of the following reasons?” Responses ranged from none to five or more times. The reasons included going to work; the grocery store or market; to get takeout from a restaurant or fast-food location; eat at a restaurant or fast-food location; the drug store or pharmacy; seek health care; check on or help care for a vulnerable person; visit friends, family, or neighbors; take a child or minor to day care or some activity, exercise, or some other outdoor activity; and attend a gathering of 10 or more. Items were summed to create a trips leaving home index with an observed range of 0-39.

#### Demographic Variables (Covariates)

*Gender* was initially assessed with five categories: male, female, transgender (identify as male), transgender (identify as female), and other. Transgender and other were collapsed.

*Political party* was assessed with four categories: Republican, Democrat, Independent, and something else.

*Race and ethnicity* were coded as White, Black, Hispanic, multiracial, and other, which included American Indian, Asian, and other.

*Income* was initially assessed with 9 strata that, for ease of presentation, were collapsed into three categories: less than US $30,000; US $30,000 to US $74,999; and US $75,000 and greater.

*Education* was initially assessed with 10 strata that were collapsed into four categories for ease of presentation: none through high school or General Educational Development, postsecondary (trade school, some college, or associate’s degree), bachelor’s degree, and advanced degree (master’s degree, doctoral degree, or professional degree).

*Religiosity* was measured with a single item: “How religious are you?” Responses ranged from not at all religious, which was coded as 1, to very religious, coded as 7.

#### Analyses

We first present sample demographic frequencies and means for key continuous independent and dependent variables. Next, bivariate correlations between the three novel predictors and the correlation of the predictors with the three COVID-19 outcomes are presented. For ease of presentation, a dichotomous variable was created using the median split for each of the three predictors. Using the median split for the three novel predictors, we next presented means for the three COVID-19 outcomes (which are all continuous), first unadjusted, with only the novel predictor in the model, then using a general linear model, adjusted for the other novel predictors along with age, gender, income, education, political party, and religiosity. Income, education, political party, and race, all categorical variables, were all dummy coded prior to entry into the multivariate model. All analyses were performed using SPSS version 25 (IBM Corp) [24]. This survey was approved by the University of Michigan’s Institutional Review Board.

### Hypotheses

The first hypothesis was that individuals that score higher on trait reactance will report fewer COVID-19 protective behaviors, higher daily excursions from their home, and lower COVID-19 knowledge.

The second hypothesis was that individuals that score higher on conspiracy beliefs regarding COVID-19 will report fewer COVID-19 protective behaviors, higher daily excursions from their home, and lower COVID-19 knowledge.

The third hypothesis was that individuals that score higher on COVID-19 apocalypse beliefs will report fewer COVID-19 protective behaviors, higher daily excursions from their home, and lower COVID-19 knowledge.

## Results

### Sample Description

The 1074 sample was 55% (n=573) female, 70% (n=723) White, 8% (n=84) Black, 9% (n=95) Hispanic, and 6% (n=65) multiracial. About 22% (n=225) of the sample had high school or lower education, and 47% (n=482) had at least a bachelor’s degree. Income distribution was about even across the three strata. With regard to political party, 29% (n=297) identified as Republican, 38% (n=395) as Democrat, 27% (n=283) as Independent, and 6% (n=61) as other. The mean for trait reactance, COVID-19 conspiracy beliefs, and COVID-19 apocalypse beliefs were 2.4, 2.9, and 2.2, respectively. Assuming a mean value of 4 or higher (corresponding to a response of agree for reactance and apocalypse items or probably true for conspiracy items) indicates a high presence of the attribute. The prevalence was 9.8% (102/1041) for apocalypse beliefs, 20.3% (214/1052) for conspiracy beliefs, and 6.9% (72/1041) for trait reactance (Table 1).

**Table 1:** Sample description (N=1074).

Variable				Participants
**Gender, n (%)**				
	Male			459 (44.3)
	Female			573 (55.4)
	Other			3 (0.1)
**Race/ethnicity, n (%)**				
	White			723 (69.9)
	Black			84 (8.1)
	Hispanic			95 (9.2)
	Multiracial			65 (6.3)
	Other			67 (6.5)
**Age (years), n (%)**				
	18-35			304 (29.5)
	36-50			263 (25.6)
	51-65			277 (26.9)
	>65			185 (18.0)
**Education, n (%)**				
	None through high school/GED^a^			225 (21.8)
	Postsecondary (trade school/some college/associate’s degree)			326 (31.6)
	Bachelor’s degree			310 (30.0)
	Advanced degree (master’s/doctoral/professional degree)			172 (16.7)
**Income (US $), n (%)**				
	<30,000			291 (28.1)
	30,000-74,999			397 (38.4)
	≥75,000			346 (33.5)
**Political party, n (%)**				
	Republican			297 (28.7)
	Democrat			395 (38.1)
	Independent			283 (27.3)
	Something else			61 (5.9)
**Variable means**				
	**COVID-19 protective behaviors index**			
		Mean (SD)	20.1 (4.6)	
		Range	5-25	
	**Trips leaving home per week**			
		Mean (SD)	6.7 (6.7)	
		Range	0-39	
	**Knowledge score**			
		Mean (SD)	26.8 (5.3)	
		Range	11-35	
	**Religiosity**			
		Mean (SD)	3.9 (2.1)	
		Range	1-7	
	**Trait reactance**			
		Mean (SD)	2.4 (1.0)	
		Range	1-5	
	**COVID-19 conspiracy beliefs**			
		Mean (SD)	2.9 (1.1)	
		Range	1-5	
	**COVID-19 apocalypse beliefs**			
		Mean (SD)	2.2 (1.1)	
		Range	1-5	

^a^GED: General Educational Development.

### Correlations

All three predictors were significantly (*P*<.01) correlated in the hypothesized direction with the three COVID-19 outcomes. Specifically, each predictor was negatively correlated with the COVID-19 protective behaviors index (range –0.10 to –0.39) and COVID-19 knowledge (range –0.42 to –0. 57). All three predictors were positively correlated with trips from home per week (range 0.27-0.31; see Table 2). The three predictors were all positively correlated (*P*<.001). Specifically, apocalypse beliefs were correlated 0.31 and 0.33 with conspiracy beliefs and reactance, respectively. Conspiracy beliefs and reactance were correlated 0.51 (data not shown).

**Table 2:** Pearson correlations of novel predictors and COVID-19 outcomes (N=1074).

Variable	COVID-19 protective behaviors	Trips leaving home per week	COVID-19 knowledge score
Trait reactance	–0.38^a^	0.31	–0.54
COVID-19 conspiracy beliefs	–0.32	0.21	–0.57
COVID-19 apocalypse beliefs	–0.09^b^	0.27	–0.42

^a^All correlations were significant with a *P* of <.001, unless otherwise indicated.

^b^*P*=.002

### Bivariate Means

Using the median split for the three predictors, the differences between the top and bottom half of participants were statistically significant across all three variables for each of the three COVID-19 outcomes (ie, protective behaviors index, trips from home, and COVID-19 knowledge). Specifically, the mean for the COVID-19 protective behaviors index and the COVID-19 knowledge scale were significantly lower in the top median compared to the bottom median for all three predictors. The mean trips from home was significantly higher in the top median compared to the bottom median for all three predictors. This model does not adjust for covariates or other novel predictors (see the unadjusted mean columns in Table 3).

In analyses accounting for all novel predictors simultaneously, plus age, gender, income, education, race, political party, and religiosity, the adjusted means remained significantly different for the top and bottom median for all outcomes except for apocalypse beliefs and the COVID-19 protective behaviors index. Thus overall, these findings were generally consistent with a priori hypotheses (see the adjusted mean columns in Table 3).

Table 3 also presents results by political affiliation. In adjusted analyses, respondents identifying as Democrat had the highest mean for the protective behaviors index and COVID-19 knowledge, whereas for trips from home, Republicans had the highest number. For all three predictors, values were highest among those identifying as Republican and lowest among those identifying as Democrat (data not shown).

Finally, we previously reported the means, using the median split of each of the three novel predictors, for each of the three outcomes. We also examined the outcome means using a threshold of 4 or higher compared to those scoring less than 4, for each of the predictors. For individuals with an average score of 4 or higher on the reactance scale, the means were 17.5, 11.2, and 22.0 for the protective behaviors index, trips from home, and knowledge score, respectively. For individuals with an average score of 4 or higher on the COVID-19 conspiracy scale, the means were 18.0, 7.8, and 23.2 for the protective behaviors index, trips from home, and knowledge score, respectively. For individuals with an average score of 4 or higher on the COVID-19 apocalypse scale, the means were 20.4, 11.0, and 23.5 for the protective behaviors index, trips from home, and knowledge score, respectively. Thus, in general, the pattern of results using the threshold of 4 or higher for the three predictors was similar to results using the median split (data not shown).

**Table 3:** Bivariate and adjusted means of COVID-19 protective behaviors, leaving home episodes, and COVID-19 knowledge by novel predictors (N=1074).

Variable			COVID-19 protective behaviors								Trips leaving home per week									COVID-19 knowledge score						
			Bivariate unadjusted, mean (SD)		*P* value		Adjusted^a^, mean (SE)		*P* value		Bivariate unadjusted, mean (SD)		*P* value		Adjusted, mean (SE)		*P* value		Bivariate unadjusted, mean (SD)			*P* value		Adjusted, mean (SE)		*P* value
**Trait reactance**					<.001				<.001				<.001				<.001					<.001				<.001
	Low^b^	21.4 (3.8)				20.9 (0.18)				5.2 (5.2)				5.9 (0.25)				28.9 (4.7)					27.8 (0.17)			
	High^c^	18.2 (5.0)				18.9 (0.22)				8.5 (7.9)				7.3 (0.30)				23.9 (4.8)					25.4 (0.20)			
**Conspiracy beliefs**					<.001				<.001				<.001				.02					<.001				<.001
	Low	21.5 (3.8)				20.9 (0.21)				5.1 (5.4)				6.0 (0.28)				29.8 (4.7)					28.7 (0.19)			
	High	18.9 (4.9)				19.4 (0.19)				7.9 (7.4)				6.9 (0.26)				24.2 (4.4)					25.2 (0.18)			
**Apocalypse beliefs**					<.001				.89				<.001				.004					<.001				<.001
	Low	20.6 (4.4)				20.1 (0.19)				5.1 (5.0)				5.9 (0.26)				28.8 (4.8)					27.6 (0.18)			
	High	19.5 (4.8)				20.1 (0.21)				8.4 (7.8)				7.1 (0.29)				24.5 (5.0)					25.9 (0.20)			
**Political party**					<.001				<.001				.19				.01					<.001				<.001
	Republican	19.3 (4.9)				19.5 (0.26)				7.1 (6.8)				7.2 (0.35)				25.4 (5.1)					26.0 (0.24)			
	Democrat	21.3 (3.8)				21.0 (0.22)				6.5 (6.9)				6.4 (0.30)				28.1 (5.5)					27.4 (0.21)			
	Independent	19.6 (4.9)				19.8 (0.25)				6.4 (6.5)				6.1 (0.34)				26.8 (5.1)					27.0 (0.23)			
	Something else	19.0 (5.2)				19.2 (0.54)				5.3 (4.1)				4.7 (0.74)				25.6 (4.4)					26.6 (0.50)			

^a^Adjusted model includes all novel predictors plus gender, race, income, education, political party, age, and religiosity.

^b^Low indicates the bottom half of the median split.

^c^High indicates the upper half of the median split.

## Discussion

### Primary Findings

Our findings indicate that three psychological factors—trait reactance, COVID-19 conspiracy beliefs, and COVID-19 apocalypse beliefs—were associated with key COVID-19 outcomes, all in the hypothesized direction. In unadjusted analyses, individuals scoring higher on trait reactance, COVID-19 conspiracy beliefs, and COVID-19 apocalypse beliefs reported lower protective behaviors and lower COVID-19 knowledge. With the exception of apocalypse beliefs and the protective behaviors index, all of these bivariate associations remained significant after adjustment for age, gender, race, income, education, religiosity, and political party. Although the three novel predictors were all correlated (ranging from 0.31 to 0.51), the magnitude of these correlations suggest that they tap largely independent dimensions of personality and attitude.

These findings have significant implications for both understanding who may adopt COVID-19 protective behavior and how intervention messages might be tailored to accommodate or counter these beliefs. With regard to reactance, our findings indicate that a subset of the US population reflexively rejects the adoption of COVID-19 protective behaviors due to a general predisposition to act in the opposite direction of authority or resist any rules or public health recommendations they feel infringe upon their personal freedom. Individuals with this trait are prone to feeling their autonomy is being threatened by government regulations or public health recommendations and will restore their freedom by rejecting the recommended behavior or counterarguing with the content and source of related messages.

With regard to conspiracy beliefs, our findings indicate that a subset of the US population believes that government officials and the media inaccurately portray the *truth* about the COVID-19 epidemic, and those who possess these beliefs are less likely to adopt COVID-19 protective behaviors. Although we did not specifically query this, it is likely this group would consider much of the mainstream media as *fake news*, making any data reports or behavioral guidelines suspect. Our findings are consistent with those of a recent study of 2501 British adults [14], which found that endorsement of COVID-19 conspiracy beliefs were significantly associated with lower self-reported adherence to recommended protective behaviors. These beliefs were also associated with general mistrust of government and authority, paranoia, vaccination conspiracy beliefs, religiosity, and climate change denial. There is a growing body of work showing an association between patterns of media consumption and endorsement of misinformation, including conspiracy beliefs. There is therefore a need to enhance media use skills and eHealth literacy in particular to help counter COVID-19–related misinformation [25,26].

Finally, individuals who believe that the COVID-19 epidemic is a signal that the end of times is nigh are less likely to adopt COVID-19 protective behaviors. Although the mechanism for this association merits elucidation, it seems plausible that individuals who believe in a coming apocalypse, particularly for those who believe they will be spared, might be less likely to adopt COVID-19 protective behaviors because, for this group, the ultimate outcome is viewed in a more positive light.

### Intervention Implications

Designing tailored communication campaigns to encourage adoption of COVID-19 protective behavior for individuals who possess the beliefs we assessed poses substantial challenges. These individuals may be particularly immune to accepting new information and yielding their beliefs. The persistence of these beliefs may in part be due to having roots in deeper psychological attributes such as paranoia, mistrust, religious fundamentalism, or hostility. Health communication professionals may apply lessons learned from countering similar beliefs around climate change and vaccine hesitancy. One lesson learned from countering antivaccination beliefs is that simply providing corrective information not only may be ineffective but also could instigate further reactance, leading to entrenchment of antivaccine attitudes [27]. Thus, messages designed for individuals prone to reactance that minimize controlling language (eg, you must or you have to) and emphasize the individual’s independence in adopting these behavioral recommendations may be more effective [28,29]. Messaging for those who possess conspiracy beliefs should similarly not assume that providing evidence contrary to these beliefs will alone alter behavior [29]. Other communication techniques such as *rolling with resistance*, a strategy used in motivational interviewing [30], which may manifest as agreeing or empathizing with some aspects of their belief system (eg, “the government is not always honest with the American people”) should be considered. Messaging for those with apocalyptic beliefs may require using religious leaders as the message source and using scripture that would support the adoption of COVID-19 protection, perhaps as an act of kindness, respect, or following God’s will. Interestingly, we found that higher scores of all three novel predictors were associated with lower COVID-19 knowledge, suggesting that lack of an accurate understanding of the virus may be driving some of these attitudes; although, it is also possible that these attributes may interfere with one’s willingness or ability to seek and absorb accurate health information. Thus, efforts to improve COVID-19 knowledge among these subgroups, in particular designing messages that mitigate inherent resistance to absorbing and yielding to new information, may be an important part of the public health messaging strategy.

This paper provides insight that may inform public health communication efforts to reduce the transmission of COVID-19 among segments of the population that may not respond to general audience messages but whose adherence to recommendations are nonetheless needed to control the pandemic.

### Limitations and Future Studies

Our data were cross-sectional, limiting directional inference. It is possible, for example, that behaviors might influence attitudes rather than the inverse. The sample was accrued entirely online, which introduces several potential sampling and response biases [31,32]. For example, our sample had a slightly lower percentage of non-Whites and a greater percentage of females and Democrats than the US population. Sample bias poses a lower threat to the validity of our findings, as we were primarily interested in exploring the association between variables rather than establishing the true prevalence of the attitudes and behaviors under study.

Our survey was administered on May 19-22, 2020. Late May appears to represent the high point of optimism about the pandemic in the United States compared to both the initial period of March and April and the second and third wave that occurred in October and November 2020. For example, according to the Gallup COVID Panel survey conducted late May 2020, the percentage of Americans who felt the COVID-19 situation was getting better was 42% [33]. Gallup had asked this question on several occasions between April and November, and this was approximately the peak for this variable for that period. The percentage feeling the situation was getting better began to drop sharply shortly after, hitting just 19% in June and remaining around only 20% through November 2020 [33]. Gallup similarly reported that, in early June, only 46% of Americans said they were very or somewhat worried about getting the virus compared to 58%-59% in July and August 2020 [34]. These data likely reflect optimism over what turned out to be short-lived falling infection and death rates during this period. These temporal patters are also reflected in data reported by the Pew Research Center, which found that in June 2020, 59% of Americans reported that they think the worst of the outbreak was still to come [35], compared to 73% who believed the worst was yet to come in April and 71% who reported the worst was yet to come in November 2020. Moreover, according to the Pew Research Center, the percent of Americans who reported wearing a mask or face covering all or most of the time when in stores and businesses over the past month was only 65% in June 2020 compared to 85% in August and 87% in November of 2020 [35]. How these background contextual factors and the specific timing of our survey administration might have impacted our results is difficult to determine. We acknowledge, however, that the pattern of our findings might have differed had we conducted our survey during times of greater overall concern about COVID-19, higher rates of mask wearing, or more stringent lockdown restrictions. For example, during periods of greater perceived risk and more strict lockdowns, it is possible that the impact of the correlates we identified might have been attenuated, as they may have been overwhelmed by a greater concern of catching the disease. Alternatively, the strength of association might have been stronger in response to the increase in perceived risk and greater restrictions. We anticipate that future studies conducted during the COVID-19 period will elucidate the generalizability of our findings across the pandemic period.

There are other potential personality and attitudinal predictors of COVID-19 protective behaviors we did not measure including trait conspiracy orientation (we measured only COVID-19 conspiracy beliefs), mistrust of government, mistrust of science, paranoia, autonomy needs, independence, hostility, intelligence, media literacy, and vaccine hesitancy. How these constructs may relate to the three we focused on merits investigation. We did not query intentions regarding uptake of a potential future COVID-19 vaccine. Understanding how the factors identified here may also be associated with COVID-19 vaccine intentions merits investigation. Future studies are needed to replicate and extend our findings, including examination of how other psychosocial and demographic factors may interact with the three predictors we studied. Additionally, work is needed to determine how best to tailor messages, both on the group and individual level, based on these constructs.
